# Pravastatin and Sarpogrelate Synergistically Ameliorate Atherosclerosis in LDLr-Knockout Mice

**DOI:** 10.1371/journal.pone.0150791

**Published:** 2016-03-07

**Authors:** Kyung-Yeon Park, Euichaul Oh, Mi-Kyoung Kwak, Hyun Sik Jun, Tae-Hwe Heo

**Affiliations:** 1 Integrated Research Institute of Pharmaceutical Sciences, College of Pharmacy, The Catholic University of Korea, Bucheon, Republic of Korea; 2 Department of Biotechnology and Bioinformatics, College of Science and Technology, Korea University, Sejong, Republic of Korea; Seoul National University College of Pharmacy, REPUBLIC OF KOREA

## Abstract

Pravastatin is a lipid-lowering agent that attenuates atherosclerosis. However, the multifactorial pathogenesis of atherosclerosis requires other drugs with different anti-atherogenic mechanisms. We chose sarpogrelate as an anti-platelet agent and a novel component of a complex drug with pravastatin due to its high potential but little information on its beneficial effects on atherosclerosis. Low-density lipoprotein receptor-knockout mice were fed a high-fat, high-cholesterol diet and treated with pravastatin alone, sarpogrelate alone, or a combination of both drugs. Although sarpogrelate alone did not significantly reduce atherosclerotic plaque areas, co-treatment with pravastatin significantly decreased aortic lesions compared to those of the pravastatin alone treated group. The combined therapy was markedly more effective than that of the single therapies in terms of foam cell formation, smooth muscle cell proliferation, and inflammatory cytokine levels. These results suggest that pravastatin and sarpogrelate combined therapy may provide a new therapeutic strategy for treating atherosclerosis.

## Introduction

Statins are a class of cholesterol lowering agents that inhibit 3-hydroxy-3-methylglutaryl coenzyme A reductase and are used to treat of cardiovascular disease (CVD) [1]. Although statins are widely used to prevent CVD, concerns have been raised about their association with an increased risk of new onset diabetes [2]. Among statins, pravastatin is recommended over other statins when statins are used in patients with atherosclerosis and who have a high risk of diabetes [3].

Atherosclerosis is a major disorder in patients with CVD, exhibiting arterial wall thickening due to accumulation of fat-laden macrophages (foam cells) and hyperplasia of fibrous and smooth muscle cells due to the complex inflammatory processes that create atherosclerotic plaques [4]. Unstable plaques are prone to rupture and induce thromboembolism and ischemia, resulting in myocardial infarction and stroke [5]. Pravastatin has been demonstrated to reduce progression of coronary atherosclerosis and myocardial infarction [6, 7]. However, a combined therapy has been investigated [8, 9] as multiple causative factors are involved in the pathogenesis of atherosclerosis. Treatment with troglitazone, an insulin enhancer, combined with pravastatin has a synergistic effect on atherosclerosis in a rabbit model [9]. Also, olmesartan and pravastatin additively reduce the development of atherosclerosis in APOE*3Leiden transgenic mice [8].

Sarpogrelate is a serotonin receptor antagonist and an anti-platelet agent widely used to prevent arterial thrombosis [10]. However, little is known about its effect on atherosclerosis, as only two studies have been published in rabbits [11, 12]. Sarpogrelate retards the progression of atherosclerosis in rabbits by upregulating endothelial nitric oxide synthase (eNOS) [12] and blood viscosity [11]. As platelets and the coagulation system are important in the progression of atherosclerosis, use of sarpogrelate with pravastatin may help prevent and treat CVD more effectively.

All patients with stable coronary artery disease require a statin and anti-platelet medication to prevent recurrent cardiovascular events [13]; thus, demonstrating the rationale for a statin/anti-platelet drug combination as atherosclerosis therapy would be valuable clinically and for research.

## Materials and Methods

### Animals

Male C57BL/6J low-density lipoprotein receptor-knockout (LDLr KO) mice (B6.129S7-*Ldlr*^*tm1Her*^/J) were obtained from Dr. Goo Taeg Oh at Ewha Women’s University (Seoul, Korea) and maintained under pathogen-free conditions in the animal facility of The Catholic University of Korea (Bucheon, Korea). The mice were fed a standard laboratory diet with free access to water. LDLr KO mice (age, 8 weeks; n = 5/group) were assigned randomly to four groups; three groups were fed a high fat, high cholesterol diet (HFD) (D12108; Research Diets Inc., New Brunswick, NJ, USA) and the fourth group was fed a standard laboratory diet (normal fat diet, NFD). Two of the HFD groups were orally administered six times weekly with sarpogrelate (10 or 50 mg/kg body weight [BW]), and the NFD and HFD with vehicle-treated groups received only 0.5% carboxymethyl cellulose (CMC; Sigma, St. Louis, MO, USA) with PBS (Welgene, Daejeon, Korea) for 20 weeks. Additional LDLr KO mice (age, 8 weeks; n = 8/group) were assigned randomly to four groups; three groups were fed a HFD and the fourth group was fed a standard laboratory diet. Two of the HFD groups were orally administered with pravastatin (40 mg/kg BW) and pravastatin (40 mg/kg BW) combined with sarpogrelate (50 mg/kg BW) five times weekly, whereas the NFD and HFD with vehicle-treated groups received only 0.5% CMC with PBS for 12 weeks. The conditions of mice were monitored everyday and there were no unexpected deaths. Mice were euthanized by an isoflurane overdose, and hearts and aortas were perfused with PBS through the left ventricle. No analgesics were administered. Animal studies were approved (2014–020) by the Department of Laboratory Animal, Institutional Animal Care and Use Committee at Sungsim campus of Catholic University of Korea (Bucheon, Korea).

### Atherosclerosis

Tissue and blood were collected from the mice after 12 or 20 weeks of feeding. The heart and aorta were perfused with PBS under anesthesia. The thoracoabdominal aorta was fixed in 4% paraformaldehyde (Sigma) to measure atherosclerotic plaque. The heart, including the aortic root, were harvested, washed, and placed in paraformaldehyde at 4°C for 24 h and embedded in Tissue CellPath OCT matrix (CellPath Ltd., Newtown, UK) for histochemistry and immunohistochemistry.

### Histochemistry and immunohistochemistry

The atherosclerotic burden of the thoracoabdominal aorta was quantified by *en face* Oil Red O-staining. The aortas were pinned on black-silicon plates in 60-mm dishes, washed twice in PBS, and dehydrated in propylene glycol at room temperature. The dish was drained and the tissue was incubated in Oil Red O dissolved in propylene glycol (Sigma) for 2 h at room temperature. Staining was developed by washing the tissue through a series of three to four dishes of 85% propylene glycol, with PBS washes after each dish. The aortas were photographed under a PBS immersion using a CCD-camera-equipped stereomicroscope (AxioCam; Carl Zeiss, Inc., Zena, Germany), the photographs were analyzed using ImageJ imaging software (National Institutes of Health, Bethesda, MD, USA), and the surface area occupied by the Oil Red O-stained lesions was determined. Serial cryostat sections (5 μm, CM1950, Leica) at the level of all three cusps within the aortic root were prepared, and the atherosclerotic lesions were analyzed at the three locations. Some of these sections were stained with Oil Red O to measure atherosclerotic plaque size. The remaining sections were used for immunohistochemical analysis of the atherosclerotic plaque composition. Air-dried sections were fixed in ice-cold acetone, blocked with 10% bovine serum albumin (Bovogen Biologicals, Keilor East VIC, Australia), and stained with anti-intercellular adhesion molecule-1 (ICAM-1; Santa Cruz Biotechnology, Santa Cruz, CA, USA), anti-monocyte/macrophage-2 (MOMA-2; Abcam, Cambridge, MA, USA), or anti-alpha smooth muscle actin antibody (α-SMA; Abcam, Cambridge, MA, USA). The sections were incubated with the appropriate HRP secondary antibody and visualized using a DAB kit (Dako, Carpentaria, CA, USA) according to the manufacturer’s instructions. All sections were counterstained with Harris hematoxylin (YD Diagnostics, Seoul, Korea). Morphometric data were obtained using a light microscope (Axio Imager D2; Carl Zeiss).

### Serum lipid analysis and cytokine assay

Blood was collected from the retro-orbit of mice in each group used for histological analysis (n = 8/group). Serum was obtained and stored at −80°C. Total cholesterol, triglycerides, high-density lipoprotein (HDL) cholesterol, and low-density lipoprotein (LDL) cholesterol were measured using a Hitachi 7020 automatic analyzer (Hitachi, Tokyo, Japan). Serum mouse interleukin (IL)-6 (Max; BioLegend Inc., San Diego, CA, USA) and mouse tumor necrosis factor (TNF) levels (ELISA ready set-go; eBioscience, San Diego, CA, USA) were measured using enzyme-linked immunoassay (ELISA) kits according to the manufacturer’s instructions.

## Results

### Sarpogrelate alone marginally and insignificantly inhibits formation of atherosclerotic plaques

**Fig 1:**
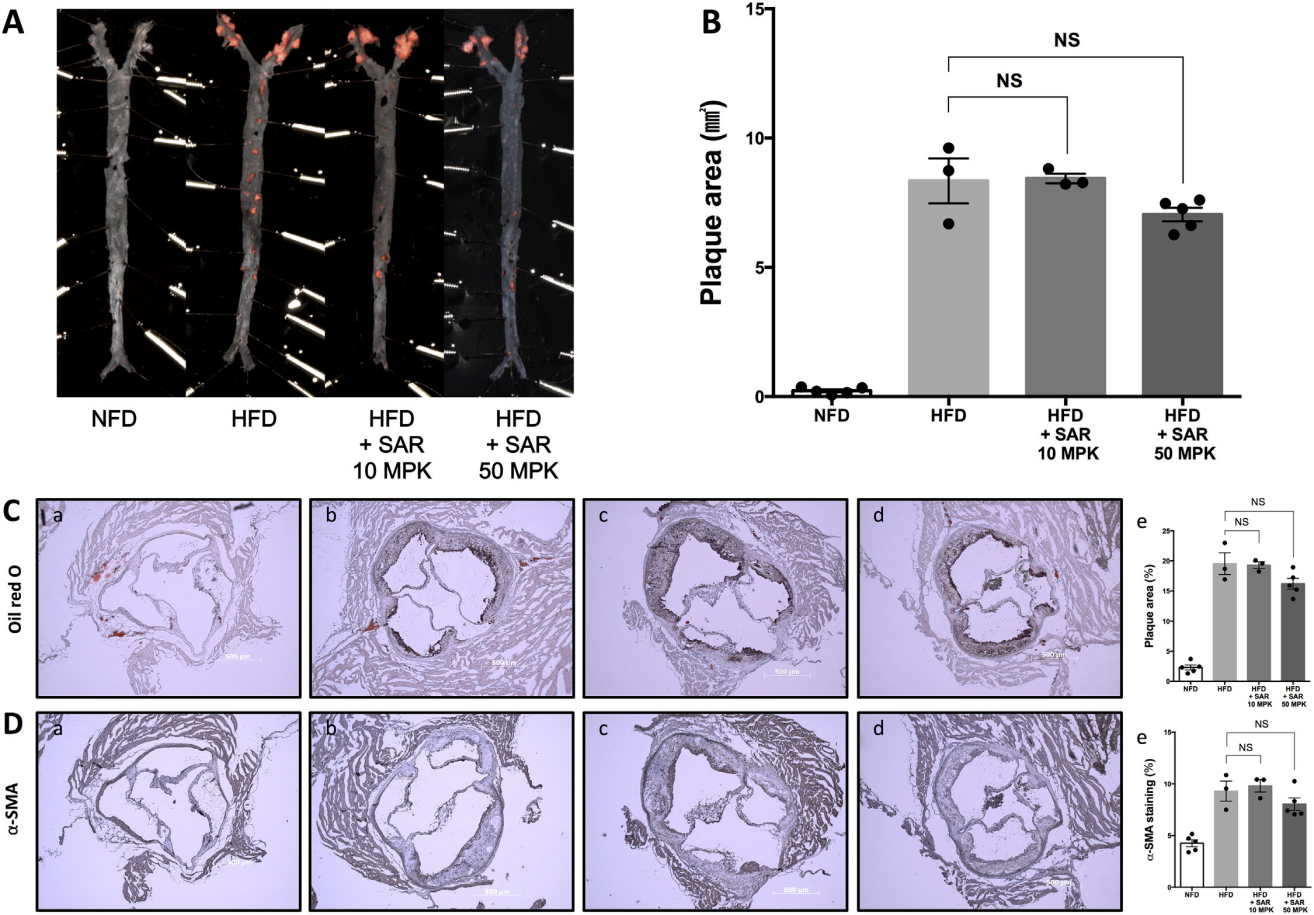
Sarpogrelate alone marginally and insignificantly reduces atherosclerotic lesions in low-density lipoprotein receptor-knockout (LDLr KO) mice. Male LDLr KO mice (8 weeks) were fed a NFD (a), a HFD (b), or a HFD with sarpogrelate (10 (c) or 50 MPK (d), orally six times weekly) for 20 weeks. (A) Representative Oil Red O-stained *en face* aortic preparation. (B) Quantification of Oil Red O-stained plaque areas. (C) Representative Oil Red O-stained atherosclerotic aortic root lesions (a, b, c, d) and quantification (e). (D) Representative alpha smooth muscle actin (α-SMA)-immunostained images of atherosclerotic plaques in aortic root lesions (a, b, c, d) and quantification (e). Scale bar represents 500 μm, Error bars represent standard errors, and significance was analyzed by Student’s *t*-test (*P < 0.05, **P < 0.01, ***P < 0.001, n = 3–5/group). MPK, mg/kg; NFD, normal fat diet; HFD, high fat diet; NS, not significant; SAR, sarpogrelate.

The anti-atherogenic effects of sarpogrelate alone were monitored using a well-established atherosclerosis model (LDLr KO mice fed a HFD). Male LDLr KO mice were fed a western-type high fat diet for 20 weeks with or without sarpogrelate treatment and plaque areas were quantified by Oil Red O staining. Sarpogrelate only slightly and insignificantly reduced plaques in the high dose-treated group (50 mg/kg, *p* = 0.0606) but not in the low dose group (10 mg/kg) (Fig 1A and 1B). Also, Oil Red O staining of atherosclerotic aortic root lesions showed slightly reduced plaque areas in the high dose-treated group (50 mg/kg, *p* = 0.0541) (Fig 1C) and α-SMA immunohistochemical staining showed marginally reduced proliferation of smooth muscle cells in the high dose group (50 mg/kg, *p* = 0.1413) (Fig 1D) but not in the low dose group (10 mg/kg). The 50 mg/kg dose of sarpogrelate was adopted for the following combination study with pravastatin.

### Combined sarpogrelate and pravastatin therapy shows a synergistic effect on the development of atherosclerosis in LDLr KO mice

**Fig 2:**
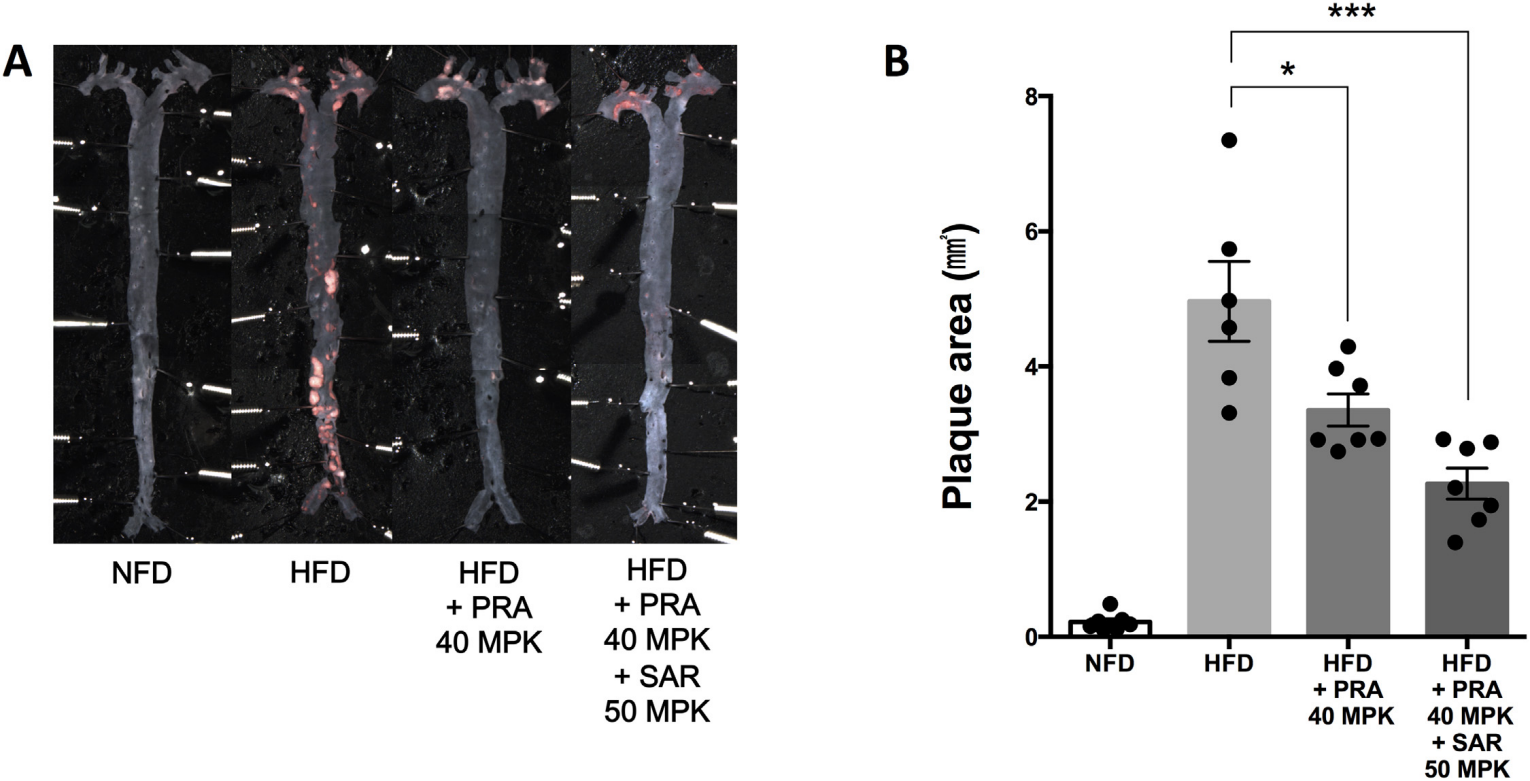
The combination of pravastatin and sarpogrelate significantly attenuates atherosclerotic lesions in low-density lipoprotein receptor-knockout (LDLr KO) mice. Male LDLr KO mice (8 weeks) were fed a NFD, a HFD, a HFD with pravastatin (40 MPK, orally five times weekly), or a HFD with a pravastatin and sarpogrelate combination (40 and 50 MPK, respectively, orally five times weekly) for 12 weeks. (A) Representative Oil Red O-stained *en face* aortic preparation. (B) Quantification of Oil Red O-stained plaque areas. Error bars represent standard error, and significance was analyzed by Student’s *t*-test (*P < 0.05, **P < 0.01, ***P < 0.001, n = 6–7/group). MPK, mg/kg; NFD, normal fat diet; HFD, high fat diet; NS, not significant; PRA, pravastatin; SAR, sarpogrelate.

Although pravastatin is effective in attenuating atherosclerosis, its activity seems to be imperfect considering the complex underlying pathogenetic mechanism of atherosclerosis. We examined whether adding sarpogrelate to a pravastatin regimen could enhance the protective effects of pravastatin alone. Oil Red O staining showed a synergistic reduction of HFD-induced plaque areas on the *en face* aortic preparations in the pravastatin (40 mg/kg) plus sarpogrelate (50 mg/kg) group compared to that in the pravastatin alone-treated group (Fig 2A and 2B).

### Markedly decreased expression of ICAM-1, infiltration of monocyte/macrophage cells, inflammatory cytokines, and reduced proliferation of smooth muscle cells contribute to the significant anti-atherosclerotic effect of combined therapy

**Fig 3:**
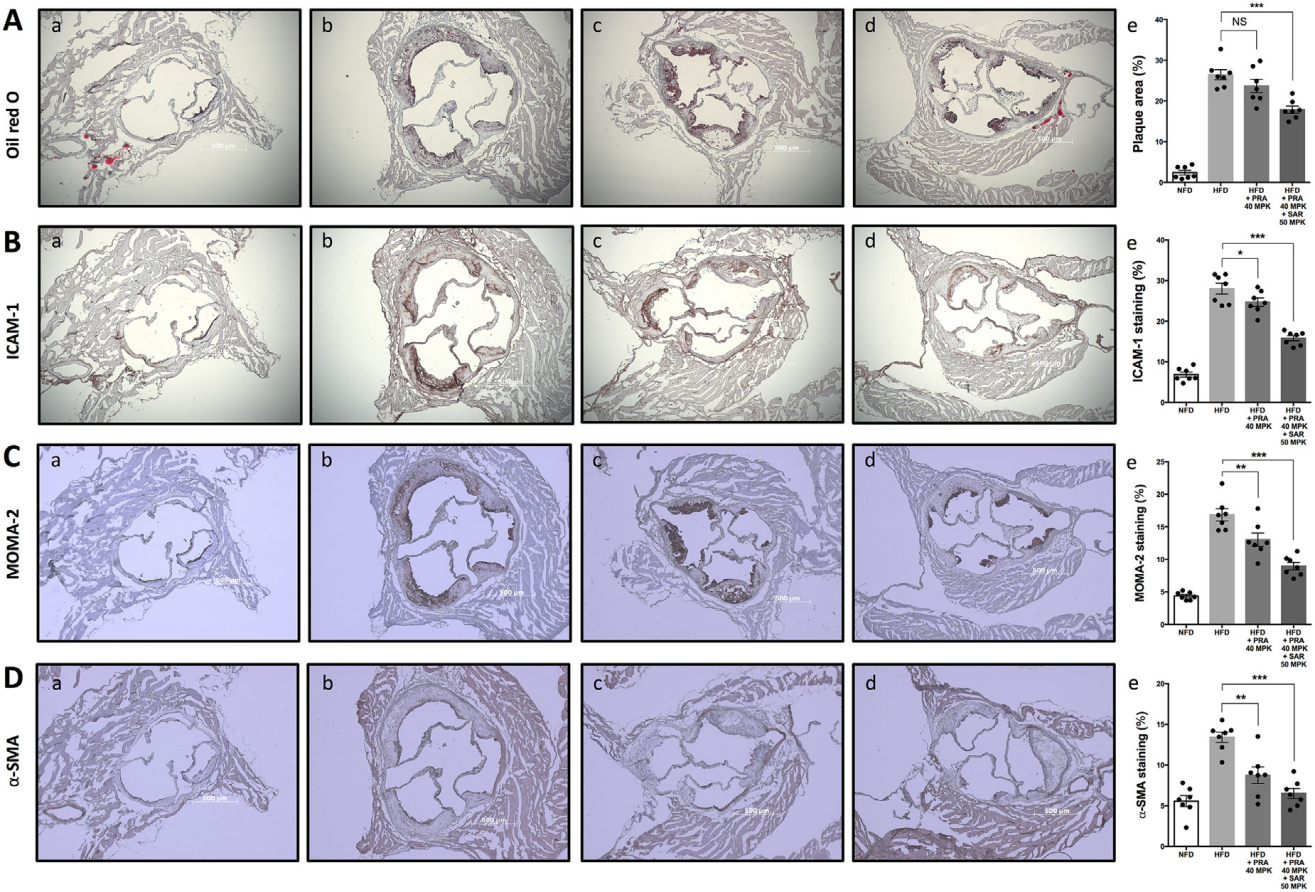
Histology of atherosclerotic aortic root lesions. Low-density lipoprotein receptor-knockout (LDLr KO) mice were fed a NFD (a), a HFD (b), a HFD with pravastatin (40 MPK) (c) or a HFD with a combination of pravastatin (40 MPK) and sarpogrelate (50 MPK) (d) for 12 weeks. Mouse aortic sections were collected and representative Oil Red O (A), intercellular adhesion molecule-1 (ICAM-1) (B), monocyte/macrophage-2 (MOMA-2) (C), and alpha smooth muscle actin (α-SMA) (D)-stained images of atherosclerotic plaques were shown and quantified (e). Scale bar represents 500 μm, Error bars represent standard errors, and significance was analyzed by Student’s *t*-test (*P < 0.05, **P < 0.01, ***P < 0.001, N = 6–7/group). MPK, mg/kg; NFD, normal fat diet; HFD, high fat diet; NS, not significant; PRA, pravastatin; SAR, sarpogrelate.

**Fig 4:**
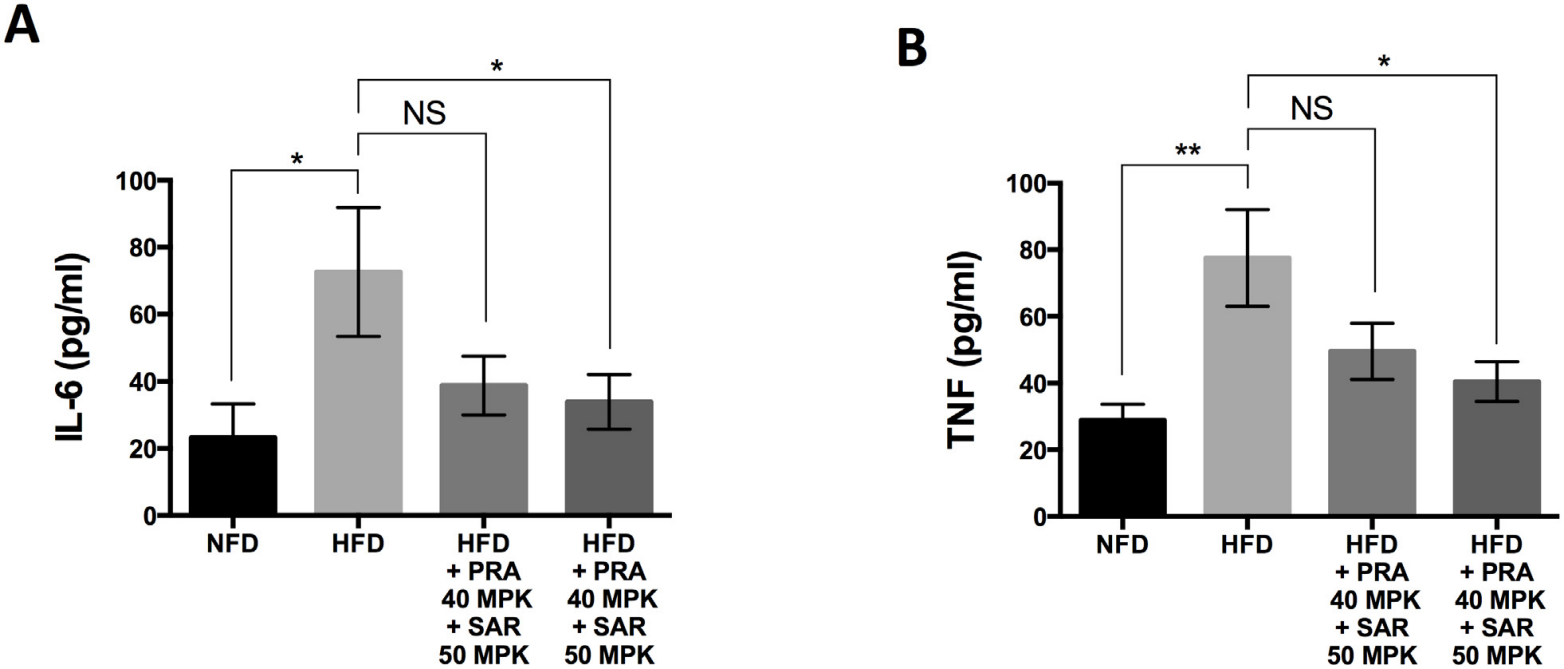
Significant suppression of inflammatory cytokines production by pravastatin/sarpogrelate combination therapy. Low-density lipoprotein receptor-knockout (LDLr KO) mice were fed a NFD, a HFD, a HFD with pravastatin (40 MPK) or a HFD with a combination of pravastatin (40 MPK) and sarpogrelate (50 MPK) for 12 weeks. Blood was collected by retro-orbital puncture before the surgery for the harvest of aorta. Serum was isolated and the levels of interleukin (IL)-6 (A) and tumor necrosis factor (TNF) (B) were measured by enzyme-linked immunosorbent assay kits according to the manufacturer’s instructions. Error bars represent standard errors, and significance was analyzed by Student’s *t*-test (*P < 0.05, **P < 0.01, ***P < 0.001, N = 6–7/group). MPK, mg/kg; NFD, normal fat diet; HFD, high fat diet; NS, not significant; PRA, pravastatin; SAR, sarpogrelate.

Next, we analyzed plaque development using Oil Red O staining, ICAM-1 expression by immunohistochemistry, monocyte/macrophage infiltration by MOMA-2, and α-SMA expression by specific antibodies in the aortic roots of LDLr KO mice that ate a NFD, HFD, or HFD with drugs. Oil Red O (Fig 3A), ICAM-1 (Fig 3B), MOMA-2 (Fig 3C), and α-SMA (Fig 3D)-stained plaque areas decreased in the combined therapy group compared to those in the pravastatin alone group. In addition, IL-6 (Fig 4A) and TNF (Fig 4B) levels decreased significantly in the combined pravastatin and sarpogrelate therapy group.

### Mean weight and plasma lipid levels of pravastatin/sarpogrelate combined group did not differ from those in the pravastatin only group

Weight gain after 12 weeks of the HFD was almost completely normalized by the pravastatin alone and combined treatments (Table 1). Lipid levels decreased significantly and similarly after 12-weeks of treatment in the pravastatin alone and combined groups, except HDL cholesterol (Table 2). The mean % change in total cholesterol level in the pravastatin group was −44% and that in the combined group was −39% compared with baseline. Similarly, the reductions in triglycerides (pravastatin: −87%, combined: −72%) and LDL cholesterol (pravastatin: −41%, combined: −36%) were comparable between the two drug-treated groups.

**Table 1:** Weights of low-density lipoprotein receptor-knockout (LDLr KO) mice fed a HFD with treatment of pravastatin or pravastatin plus sarpogrelate.

Weight (g)	NFD	HFD	HFD + PRA 40 mg/kg	HFD + PRA 40 mg/kg + SAR 50 mg/kg
0 Week	23.44 ± 0.74	22.92 ± 0.87	23.21 ± 0.63	23.17 ± 0.73
12 Week	27.72 ± 1.11	38.99 ± 1.64	28.94 ± 1.92 ***	28.74 ± 1.44 ***

Data represented as mean ± standard error and significance was analyzed by Student’s *t*-test compared (*** denotes significant difference to HFD group, ***P < 0.001, n = 7–8/group).

Abbreviations: NFD, normal fat diet; HFD, high fat diet; SAR, sarpogrelate; PRA, pravastatin

**Table 2:** Serum lipid analysis of low-density lipoprotein receptor-knockout (LDLr KO) mice fed a HFD with treatment of pravastatin or pravastatin plus sarpogrelate.

Milligrams/deciliter	NFD	HFD	HFD + PRA 40 mg/kg	HFD + PRA 40 mg/kg + SAR 50 mg/kg
Total cholesterol	351.1 ± 17.67	2132 ± 111.1	1353 ± 104.3 ***	1446 ± 84.13 ***
Triglycerides	113.3 ± 11.94	1048 ± 195.1	232.0 ± 33.29 ***	377.1 ± 18.00 **
HDL cholesterol	100.7 ± 3.496	87.25 ± 5.593	89.50 ± 2.693	84.50 ± 3.813
LDL cholesterol	111.1 ± 7.818	787.5 ± 40.64	512.5 ± 32.14 **	542.3 ± 34.37 **

Data represented as mean ± standard error and significance was analyzed by Student’s *t*-test (**,*** denote significant difference to HFD group, **P < 0.01, ***P < 0.001, n = 7–8/group).

Abbreviations: NFD, normal fat diet; HFD, high fat diet; SAR, sarpogrelate; PRA, pravastatin

## Discussion

Despite the efficacy of statins for atherosclerosis, statin monotherapy is insufficient to reach the treatment goals of some patients due to their side effects and the multifactorial pathogenesis of atherosclerosis, including platelet activation [14]. Although many clinical trials have compared statin monotherapy to combined therapy with statins and other lipid-modifying agents, few data are available regarding combined therapy of statin with a non-lipid-modifying agent. In the present study, we evaluated the effect of combined pravastatin and sarpogrelate therapy in LDLr KO mice for the first time.

Anti-platelet agents have been proposed to be effective in preventing cardiovascular events [15]. Specifically, sarpogrelate [11], cilostazol [16], and clopidogrel [17] have beneficial effects in the prevention of atherosclerosis in a rabbit model. In our study, treatment with sarpogrelate alone resulted in no reduction in arterial wall plaque formation at a dose of 10 mg/kg, and a marginal and insignificant reduction was observed at 50 mg/kg (Fig 1A and 1B). Similarly, Oil Red O staining and immunohistochemical staining for α-SMA did not show a dramatic regression of plaque areas and smooth muscle cell proliferation (Fig 1C and 1D). Although sarpogrelate alone did not show dramatic therapeutic effects against progression of atherosclerosis, we hypothesized that adding sarpogrelate to statin therapy could maximize the anti-atherosclerotic effects of both drugs.

Pravastatin has a low risk of new onset diabetes compared with that of atorvastatin, rosuvastatin, and simvastatin [3]. Except for a report regarding the synergistic effects of pravastatin combined with troglitazone, which is an insulin action enhancer [9], little clinical and basic data are available about combinations with pravastatin. In our study, the pravastatin/sarpogrelate combination markedly reduced development of atherosclerosis as demonstrated by decreased plaque area, histology, and inflammatory markers, which are new observations.

Pravastatin monotherapy reduces the progression of plaque lesions by decreasing plasma lipid levels and anti-inflammatory properties [8], including suppression of IL-6 [18] and interferon-γ expression [4]. The anti-atherogenic effects of sarpogrelate are associated with inhibiting platelet aggregation, upregulating eNOS, and suppressing smooth muscle cell proliferation [10, 12]. Although Xu et al. demonstrated significantly reduced plasma cholesterol and triglyceride levels in rabbits [11], sarpogrelate did not affect lipid levels in our LDLr KO mice (Table 3). Our results suggest that beneficial effects of combined therapy for treating atherosclerosis are attributed to the anti-proliferative effects on macrophages and smooth muscle cells and the suppression of inflammatory cytokines production by a combined drug as well as the lipid-lowering effects of pravastatin.

**Table 3:** Serum lipid analysis of low-density lipoprotein receptor-knockout (LDLr KO) mice fed a HFD with treatment of sarpogrelate.

Milligrams/deciliter	NFD	HFD	HFD + SAR 10 mg/kg	HFD + SAR 50 mg/kg
Total cholesterol	374.4 ± 23.52	1239 ± 92.21	1308 ± 77.87	1399 ± 6.960
Triglycerides	105.2 ± 13.63	865.5 ± 94.05	874.0 ± 113.3	799.2 ± 82.38
HDL cholesterol	88.40 ± 3.487	86.00 ± 2.944	90.00 ± 7.257	93.20 ± 3.980
LDL cholesterol	97.60 ± 7.026	454.7 ± 34.59	510.7 ± 34.14	559.3 ± 16.51*

Data represented as mean ± standard error and significance was analyzed by Student’s *t*-test (* denotes significant difference to HFD group, *P < 0.05, n = 3–5/group).

Abbreviations: NFD, normal fat diet; HFD, high fat diet; SAR, sarpogrelate

Our results show that atherosclerotic plaque areas decreased substantially in response to the pravastatin and sarpogrelate combined therapy compared with those of monotherapy with either drug. It appears that the superior effects of the combined therapy are involved in the anti-inflammatory and anti-proliferative effects of both pravastatin and sarpogrelate.

## Abbreviations

CVD: cardiovascular disease
LDLr KO: low-density lipoprotein receptor-knockout
TNF: tumor necrosis factor
NFD: normal fat diet
HFD: high fat diet

## References

[1] Collaboration PS. Blood cholesterol and vascular mortality by age, sex, and blood pressure: a meta-analysis of individual data from 61 prospective studies with 55 000 vascular deaths. The Lancet. 2007;370(9602):1829–39. 10.1016/S0140-6736(07)61778-4 18061058

[2] Chogtu B, Magazine R, Bairy KL. Statin use and risk of diabetes mellitus. World J Diabetes. 2015;6(2):352–7. 10.4239/wjd.v6.i2.352 25789118 PMC4360430

[3] Carter AA, Gomes T, Camacho X, Juurlink DN, Shah BR, Mamdani MM. Risk of incident diabetes among patients treated with statins: population based study. BMJ. 2013;346:f2610 10.1136/bmj.f2610 23704171 PMC3662830

[4] Zhou X-X, Gao P-J, Sun B-G. Pravastatin attenuates interferon-gamma action via modulation of STAT1 to prevent aortic atherosclerosis in apolipoprotein E-knockout mice. Clin Exp Pharmacol Physiol. 2009;36(4):373–9. 10.1111/j.1440-1681.2008.05067.x 19018808

[5] Finn AV, Nakano M, Narula J, Kolodgie FD, Virmani R. Concept of Vulnerable/Unstable Plaque. Arteriosclerosis, Thrombosis, and Vascular Biology. 2010;30(7):1282–92. 10.1161/ATVBAHA.108.179739 20554950

[6] Daida H, Ouchi Y, Saito Y, Yamada N, Nishide T, Mokuno H, et al Preventing angiographic progression of coronary atherosclerosis with pravastatin. J Atheroscler Thromb. 2003;10(1):25–31. 12621161 10.5551/jat.10.25

[7] Pitt B, Mancini GBJ, Ellis SG, Rosman HS, Park J-S, Mcgovern ME. Pravastatin limitation of atherosclerosis in the coronary arteries (PLAC I): Reduction in atherosclerosis progression and clinical events. J Am Coll Cardiol. 1995;26(5):1133–9. 7594023 10.1016/0735-1097(95)00301-0

[8] van der Hoorn JWA, Kleemann R, Havekes LM, Kooistra T, Princen HMG, Jukema JW. Olmesartan and pravastatin additively reduce development of atherosclerosis in APOE*3Leiden transgenic mice. J Hypertens. 2007;25(12):2454–62. 17984667 10.1097/HJH.0b013e3282ef79f7

[9] Shiomi M, Ito T, Tsukada T, Tsujita Y, Horikoshi H. Combination treatment with troglitazone, an insulin action enhancer, and pravastatin, an inhibitor of HMG-CoA reductase, shows a synergistic effect on atherosclerosis of WHHL rabbits. Atherosclerosis. 1999;142(2):345–53. 10030386 10.1016/s0021-9150(98)00259-7

[10] Saini HK, Takeda N, Goyal RK, Kumamoto H, Arneja AS, Dhalla NS. Therapeutic potentials of sarpogrelate in cardiovascular disease. Cardiovasc Drug Rev. 2004;22(1):27–54. 14978517 10.1111/j.1527-3466.2004.tb00130.x

[11] Xu Y-J, Zhang M, Ji L, Elimban V, Chen L, Dhalla NS. Suppression of high lipid diet induced by atherosclerosis sarpogrelate. J Cell Mol Med. 2012;16(10):2394–400. 10.1111/j.1582-4934.2012.01554.x 22348587 PMC3823433

[12] Hayashi T, Sumi D, Matsui-Hirai H, Fukatsu A, Arockia Rani P J, Kano H, et al Sarpogrelate HCl, a selective 5-HT2A antagonist, retards the progression of atherosclerosis through a novel mechanism. Atherosclerosis. 2003;168(1):23–31. 12732383 10.1016/s0021-9150(03)00054-6

[13] Pflieger M, Winslow BT, Mills K, Dauber IM. Medical management of stable coronary artery disease. Am Fam Physician. 2011;83(7):819–26. 21524048

[14] Monroe AK, Gudzune KA, Sharma R, Chelladurai Y, Ranasinghe PD, Ansari MT, et al Combination Therapy Versus Intensification of Statin Monotherapy: An Update. AHRQ Comparative Effectiveness Reviews. 2014. 24624458

[15] Kim S-M, Cho K-I. Impact of cilostazol on the progression of carotid atherosclerosis in patients with retinal vascular occlusion. Cardiovasc Ther. 2013;31(6):e94–e101. 10.1111/1755-5922.12042 24034133

[16] da Rosa MP, Baroni GV, Portal VL. Cilostazol, a phosphodiesterase III inhibitor: future perspectives in atherosclerosis. Arq Bras Cardiol. 2006;87(5):e222–6. 17396201 10.1590/s0066-782x2006001800035

[17] Li M, Zhang Y, Ren H, Zhang Y, Zhu X. Effect of clopidogrel on the inflammatory progression of early atherosclerosis in rabbits model. Atherosclerosis. 2007;194(2):348–56. 17156785 10.1016/j.atherosclerosis.2006.11.006

[18] Zhou X, Li D, Yan W, Li W. Pravastatin prevents aortic atherosclerosis via modulation of signal transduction and activation of transcription 3 (STAT3) to attenuate interleukin-6 (IL-6) action in ApoE knockout mice. IJMS. 2008;9(11):2253–64. 10.3390/ijms9112253 19330073 PMC2635628

